# Transitioning to Del Nido cardioplegia for all-comers: the next switching gear?

**DOI:** 10.1186/s12872-020-01506-0

**Published:** 2020-05-08

**Authors:** Mohamed Marzouk, Valerie Lafreniere-Bessi, Stephanie Dionne, Serge Simard, Christian Pigeon, François Dagenais, Niv Ad, Frederic Jacques

**Affiliations:** 1 Service of Cardiac Surgery, Institut universitaire de cardiologie et de pneumologie de Québec-IUCPQ, Université Laval, Quebec City, QC G1V 4G5 Canada; 2 Biostatistics, Institut universitaire de cardiologie et de pneumologie de Québec-IUCPQ, Université Laval, Québec, QC Canada; 3 Department of Cardiovascular and Thoracic Surgery, West Virginia University, Morgantown, WV USA

## Abstract

**Background:**

Exclusive use of Del Nido cardioplegia administration in all adult patients undergoing cardiac surgery has been studied for operative, postoperative and myocardial protection outcomes.

**Methods:**

From November 2016 to October 2017, Del Nido cardioplegia was used in 131 consecutive patients (DN group). Using a propensity score, DN group was compared to 251 patients having received intermittent cold blood cardioplegia (CB group).

**Results:**

Preoperative characteristics were similar in DN and CB groups. Operative outcomes were statistically different (*p* < 0.0001): cardiopulmonary bypass (CPB) time (DN 105.9 ± 46.5, CB 131.2 ± 38.8); aortic cross-clamp time (DN 80.8 ± 35.5, CB 102.2 ± 31.3); operative time (DN 203.1 ± 65.0, CB 241.5 ± 54.7); total cardioplegia volume (DN 1328 ± 879, CB 3773 ± 1226); and peak glycemia on CPB (DN 8.2 ± 2.3, CB 9.0 ± 1.8). No statistical differences were noted in intensive care unit stay, hospital stay and hospital death. Myocardial protection outcomes were similar: discharge left ventricular ejection fraction (DN 52 ± 11, CB 51 ± 10); Troponin levels at the end of the surgery (DN 871 ± 1623, CB 1958 ± 854), day 1 (DN 853 ± 1139, CB 993 ± 8234) and day 4 (DN 442 ± 540, CB 463 ± 317).

**Conclusion:**

Del Nido cardioplegia use in all adult cardiac surgeries is associated with improved surgical efficiency. The design of larger trials including adults combined cardiac procedures and emergencies is needed.

## Background

Myocardial protection is the cornerstone of cardiac surgery [1]. The wide spectrum of strategies for myocardial protection ranges from the beating heart to diastolic arrest with cardioplegia. Cardioplegic arrest has been the preferred method of myocardial protection in most centers [2]. The ideal cardioplegia solution and administration regimen is still to be found [3].

Del Nido cardioplegia, extensively used in pediatric cardiac surgery, provides a bloodless quiescent operative field for a longer period than other cardioplegia solutions [4, 5]. This may improve the surgical flow and the patient outcomes.

In the last decade, Del Nido cardioplegia was gradually integrated into adult cardiac surgery surgical practice. Nevertheless, concerns were raised for use its use in the acquired cardiovascular disease population, partly because safety and efficacy data is missing [3]. So far, a number of small reports focused on a narrow portion of the practice and have been reassuring: 1) adult congenital [6], primary valve procedure [7–11], iterative valvular procedure [12], primary CABG [13–15], iterative CABG [16], and combined CABG and valvular procedures [17, 18].

This study reports the transition to Del Nido cardioplegia use among all adult cardiac surgical patients. Del Nido cardioplegia was compared to intermittent cold blood cardioplegia in terms of operative, postoperative as well as myocardial protection outcomes.

## Methods

### Patient selection

From November 2016 to October 2017, Del Nido cardioplegia was used in 131 consecutive patients (DN group) and compared using a propensity score to 251 patients who had received intermittent cold blood cardioplegia (CB group) and operated during the immediate period prior. All patients were operated by one surgeon (FJ). All cases were included in the study with no exception.

### Cardioplegia administration

#### In the CB group

Cold blood cardioplegia was administrated at 4 °C in antegrade and/or retrograde. Oxygenated blood from patient was withdrawn (1 L) after CBP start. An addition of 200 meq/100 mL of KCl with 75 mg/100 mL of Magnesium and 50 mg of Lidocaine for each 600 mL of cardioplegia solution was added. A volume of 600 mL of cold blood solution was delivered for the induction dose. Maintenance doses were given every 15–20 min with a volume of 300 mL and half of the components of the induction solution. A “hot-shot” reperfusion dose before unclamping the aorta. Moderate systemic hypothermia reached 32 °C in isolated aortic valve replacement and in coronary bypass grafting (CABG) with less than 3 distal anastomoses, whereas a temperature of 30 °C was reached in other cases.

#### In the DN group

Del Nido cardioplegia was delivered at 4 °C with a 20% blood mixture as described by Matte et al [4]. Diastolic arrest was achieved with 1 L antegrade administration, if not, retrograde administration was used in adjunct. Redosing was performed with aortic cross-clamp time greater than 90 min; with 500 mL or 1000 mL, depending on the expected remaining surgical time. “Hot-shot” was not given in this group. All patients were put under moderate or deep hypothermia to balance the increase interval-redosing period.

Pericardial ice sludge was not used as this myocardial protection strategy is also linked to diaphragm dysfunction in obese or chronic obstructive pulmonary disease patients [19].

### Statistical analysis

The representative measures use standard descriptive statistics to describe the data; absolute and relative frequencies for categorical variables and mean ± standard deviation (SD) or median with interquartile range for continuous data according to the variable distribution. Statistical significance of differences between unmatched patient groups was tested using the Fisher’s exact test for categorical variables; the Student’s t-test or the Wilcoxon rank-sum test was performed for continuous variables. Using the residuals from the one-way statistical modeling of continuous variables, the normality assumption was verified with the Shapiro-Wilk test and the Brown and Forsythe’s variation of Levene’s test statistic was used to verify the homogeneity of variances.

A continuous propensity score analysis was performed. The likelihood of having a Del Nido cardioplegia was calculated for each patient by use of a logistic regression analysis that identified variables independently associated with Del Nido cardioplegia and the outcome. Variables included in the logistic regression analysis were the ones reported in the baseline characteristics table. Continuous variables were checked for the assumption of linearity in the logit and the graphical representations suggesting linear relationships. The variables were selected only if they maximized the within sample correct prediction rates. Interactions between variables were allowed only if it was supported clinically and statistically (*p* < 0.20). After model building, to assess the goodness-of-fit of the model, the Hosmer-Lemeshow test was performed, resulting with the statistic of Chi-Square = 4.08 with df = 7 and *p* = 0.7700, indicating that the final model fitted quite well [20]. Propensity scores were finally developed based on all this covariates: atrial fibrillation, stroke, unstable angina, surgical categories. None of them are continuous variables. Thereafter, matched set on the propensity score without replacement of case and control subjects (many-to many or full matching) was performed using the Greedy matching algorithm to provide power to the propensity score. A 1:1 match yielded similar results but with less confidence (results not shown). Full matching involved the formation of strata, consisting of either one case subject (Del Nido cardioplegia) and at least one control subject or one control subject and at least one Del Nido cardioplegia subject. Subject with a Del Nido cardioplegia procedure were considered sequentially. Best match are case subjects matched to control subjects whose score equals that of the treated subjects to at least the sixth digit. When all matches at the sixth digit are exhausted, the process begins again until matches are performed on the first digit of the propensity score. At the end, if a treated subject cannot be matched to any subject of the other group on the first digit of the propensity score, then the subject is discarded from the matched analysis. At the sixth digit, 130 out of Del Nido cardioplegia patients (99.2%) were fully matched to 228 intermittent cold blood cardioplegia patients (13 stratifications). After full matching, statistical analyses used a weighting approach, considering the clustering of subjects within each stratification. Del Nido cardioplegia subjects received a weight of 1. Intermittent cold blood cardioplegia individuals in stratification received a weight proportional to case subjects divided by the number of Del Nido cardioplegia subject in the strata. The sum of the intermittent cold blood cardioplegia subject weights was scaled to equal the total number of matched Del Nido cardioplegia subjects (130). Continuous variables were expressed as weighted mean ± SD. Categorical variables were expressed using proportions and analysed with a linear model and a logit link function adjusted for stratification (blocking factor) and weight [21].

To compare observed versus predicted means of length of stay and intensive care unit (ICU) length of stay during November 1st 2016 and November 1st 2017, a linear regression model with quarterly values as the only predictor was used to model the means observed between February 1st 2015 and August 1st 2016. The statistical models were used to predict quarterly the mean values for the periods 2016–2017.

Statistical significance was present when the two-tailed *p* value < 0.05. Analyses were performed using SAS version 9.4 (SAS Institute Inc., Cary, NC). The institutional research ethics board approved the study.

## Results

### Patient population

Demographics of all-comer patients are detailed in Table 1. Preoperative clinical characteristics were similar except for prior to matching, there were more cases with history of atrial fibrillation or redo surgeries in DN group and there were more cases with severe angina or non-elective surgeries in CB group. The proportion of patients with left ventricular ejection fraction (LVEF) less than 35% was 7.8% in DN group and 9.6% in CB group (*p* = 0.62). The LV mass index was 96 ± 29 mm in the DN group and 97 ± 22 mm in the CB group (*p* = 0.69). Moderate or severe aortic insufficiency was reported in 8.5% of patients in DN group and in 7.4% of patients in CB group (*p* = 0.74). Surgical procedures are outlined in Table 2. Matched groups did not show any significant difference in terms of types of procedure. Six cases of aortic dissections were included, two of which were done in DN group.

**Table 1:** Preoperative patient characteristics

		UNMATCHED			MATCHED	
	DN (*n* = 131) No.(%) or mean ± SD(MED)	CB (*n* = 251) No.(%) or mean ± SD(MED)	p value	DN (*n* = 130) % or mean ± SD	CB (*n* = 228) % or mean ± SD	SMD
Age (years)	65.8 ± 12.9(68)	65.8 ± 11.3(67)	0.99	65.8 ± 12.9	65.1 ± 9.2	0.06248
Sex F	35(26.7)	73(29.1)	0.71	26.2	32.7	− 0.14296
body mass index (kg/m^2^)	28.4 ± 5.7	29.0 ± 5.8	0.35	28.4 ± 5.8	28.9 ± 4.6	−0.09552
Diabetes	37(28.2)	34(33.5)	0.35	28.5	28.8	−0.00664
Hypertension	92(70.2)	192(76.5)	0.21	70.0	72.5	−0.05526
COPD	21(16.9)	29(11.6)	0.26	15.4	14.8	0.01676
Stroke	5(3.8)	21(8.4)	0.13	3.1	3.1	0
CKD and or dialysis	9(6.9)	10(4.0)	0.22	6.2	4.5	0.07560
peripheral vascular disease	9(6.9)	31(12.4)	0.11	6.9	11.3	−0.15343
Atrial Fibrillation	38(29)	43(17.1)	0.008	28.5	28.5	0
NYHA III or IV/IV	34(26.0)	65(25.9)	1.0	26.2	31.1	−0.10854
Angina CCS >3	30(22.9)	83(33.1)	0.04	23.1	20.6	0.06053
Unstable angina	10(7.6)	51(20.3)	0.0011	7.7	7.7	0
Non-elective	15(11.5)	58(23.1)	0.006	11.5	14.8	−0.09777
Redo	19(14.5)	18(7.2)	0.02	13.9	12.2	0.05048
LVEF	54 ± 11	53 ± 11	0.62	54 ± 11	53 ± 9	0.09950
LVEF <50%	27(20.9)	59(23.6)	0.60	21.1	23.4	−0.05532
LVEF <35%	10(7.8)	20(8.0)	1.0	7.8	9.6	−0.06390
Aortic Insufficiency >2/4	11(8.5)	19(7.7)	0.84	8.5	7.4	0.04067
LV Mass Index	96 ± 29(92)	96 ± 29(92)	0.95	96 ± 29	97 ± 22	−0.03885
Parsonnet Score	2.9 ± 3.7	2.7 ± 3	0.59	2.7 ± 3.4	2.9 ± 2.3	−0.06890
Euroscore II	5.2 ± 7.1	5.3 ± 7.1	0.92	5.1 ± 7.0	5.8 ± 5.5	−0.11120

*CCS* Canadian Cardiovascular Society, *CKD* chronic kidney disease (creatinine>2 mg/dl), *COPD* chronic obstructive lung disease, *LVEF* left ventricular ejection fraction, *NYHA* New York Heart Association

**Table 2:** Surgical categories

		UNMATCHED			MATCHED	
	DN (n = 131) No.(%)	CB (n = 251) No.(%)	p value	DN (n = 130) %	CB (n = 228) %	SMD
Isolated CABG	62(47.3)	145(57.8)	0.006	47.7	58.8	−0.22386
Valvular procedures	29(22.1)	27(10.8)		21.5	11.8	0.26262
CABG+Valvular procedures	15(11.5)	43(17.1)		11.5	14.5	−0.08929
Others	25(19.0)	36(14.3)		19.2	14.9	0.11453

### Operative

Operative characteristics and CBP data are described in Table 3. Surgical times were shorter in DN group (*p* < 0.0001). Increased statistical significance was found with CABG and combined procedures (CABG+ valves) and to a lesser degree with valve only procedures. Valve only procedures had greater statistical impact for time spared with multiple compared to single valve procedures. Antegrade cardioplegia was administered for most cases except for aortic dissections. A retrograde route was used in 13.1% of surgeries in DN group and in 38.0% of surgeries in CB group (*p* < 0.0001). The total cardioplegia volume given during surgery in DN group was less than in CB group (1328 ± 879 mL vs 3773 ± 1226 mL, p < 0.0001). Defibrillation after unclamping the aorta was significantly lower in the DN group (16.9% vs 32.4%, *p* = 0.01). The average peak blood sugar during CPB was lower in DN group (8.2 ± 2.3 vs 9.0 ± 1.8, *p* = 0.001). The proportion of patient with a blood sugar higher than 10 mmol/L at the end of CPB and receiving blood transfusions during CPB were similar in both groups.

**Table 3:** Operative characteristics and cardiopulmonary bypass (CBP) data

		UNMATCHED			MATCHED	
	DN (n = 131) No.(%) or mean ± SD	CB (n = 251) No.(%) or mean ± SD	p value	DN (n = 130) % or mean ± SD	CB (n = 228) % or mean ± SD	p value
Cross-Clamp Time (min)	81.1 ± 35.6	99.4 ± 41.2	< 0.0001	80.8 ± 35.5	102.2 ± 31.3	<0.0001
Isolated CABG	64.6 ± 19.2	80.1 ± 24.1	< 0.0001	65.9 ± 4.6	85.8 ± 4.6	< 0.0001
Valvular procedures	76.8 ± 30.3	82.0 ± 31.2	0.60	88.8 ± 17.9	110.3 ± 17.9	0.02
CABG+Valvular procedures	104.3 ± 31.0	134.8 ± 41.4	0.05	106.7 ± 7.7	146.0 ± 7.7	0.001
Others	95.3 ± 43.1	120.7 ± 45.6	0.002	113.8 ± 14.3	128.9 ± 14.0	0.24
Isolated valve	91.9 ± 41.4(85) *N* = 50/61	122.8 ± 43.2(123) *N* = 79/91	< 0.0001	104.3 ± 9.4	127.9 ± 9.2	0.0005
Multiple valve	126.7 ± 31.7(120) *N* = 11/61	174.0 ± 43.2(179) *N* = 12/91	0.0074	126.7 ± 10.9	167.6 ± 11.6	0.0179
CBP time (min)	106.3 ± 46.6	125.1 ± 49.6	0.0004	105.9 ± 46.5	131.2 ± 38.8	<0.0001
Isolated CABG	81.6 ± 22.0	99.2 ± 28.0	0.0001	84.9 ± 7.6	109.3 ± 7.6	< 0.0001
Valvular procedures	104.3 ± 34.1	113.1 ± 49.3	0.51	112.4 ± 16.1	136.7 ± 16.1	0.03
CABG+Valvular procedures	127.8 ± 5.5	164.0 ± 47.3	0.04	132.4 ± 10.0	177.3 ± 10.0	0.001
Others	127.6 ± 58.3	153.8 ± 52.4	0.008	149.3 ± 11.8	166.3 ± 11.8	0.31
Isolated valve	124.0 ± 54.1(118) N = 50/61	154.1 ± 51.7(152) N = 79/91	0.0020	137.7 ± 11.2	160.3 ± 11.0	0.0089
Multiple valve	157.7 ± 43.3(146) N = 11/61	210.6 ± 46.4(206) N = 12/91	0.0104	157.7 ± 12.9	205.4 ± 13.7	0.0192
Length of surgery ‘SkinSkin’ (min)	204.0 ± 65.5	236.4 ± 67.2	< 0.0001	203.1 ± 65.0	241.5 ± 54.7	<0.0001
Isolated CABG	178.5 ± 32.7	212.0 ± 46.7	< 0.0001	183.7 ± 8.2	224.2 ± 8.2	< 0.0001
Valvular procedures	198.8 ± 63.3	211.4 ± 83.7	0.59	205.7 ± 25.3	229.3 ± 25.3	0.19
CABG+Valvular procedures	238.0 ± 94.5	270.8 ± 63.0	0.25	235.3 ± 15.2	286.2 ± 15.2	0.02
Others	225.2 ± 75.9	267.0 ± 73.9	0.002	251.2 ± 15.2	293.4 ± 15.2	0.05
Isolated valve	224.3 ± 85.6(215) N = 50/61	262.5 ± 76.1(255) N = 79/91	0.0092	240.0 ± 16.5	271.2 ± 16.2	0.0200
Multiple valve	246.4 ± 53.1(230) N = 11/61	304.9 ± 95.0(324) N = 12/91	0.0861	246.4 ± 22.3	299.9 ± 23.7	0.1143
Retrograde cardioplegia	17(13.0)	76(31.3)	0.0001	13.1	38.0	<0.0001
Antegrade cardioplegia	130(99.2)	249(99.2)	1.0	99.2	98.9	0.81
Total cardioplegia dose (mL)^a^	1331 ± 876	3719 ± 1571	< 0.0001	1328 ± 879	3773 ± 1226	<0.0001
Minimal temperature on CPB(°C)	30.4 ± 3.2	31.1 ± 3.4	0.005	30.5 ± 3.2	30.8 ± 2.8	0.28
Glucose peak on CPB (mmol/l)	8.2 ± 2.3	8.8 ± 2.4	0.011	8.2 ± 2.3	9.0 ± 1.8	0.001
Glucose end CBP (mmol/l)	8.4 ± 2.6	8.2 ± 2.4	0.10	8.4 ± 2.7	8.9 ± 1.9	0.06
Glucose >10 end of CBP (mmol/l)	24(20.0)	62(26.2)	0.24	20.2	24.2	0.46
Transfusion in OR	48(36.6)	104(41.4)	0.38	36.2	42.7	0.27
Intraoperative Defibrillation	21 (16)	69 (27.5)	0.02	16.9	32.4	0.01

^a^All cardioplegia volumes shown in this text and tables are the total volume given to the patient, including the crystalloid and the blood components

### Postoperative

**Fig. 1:**
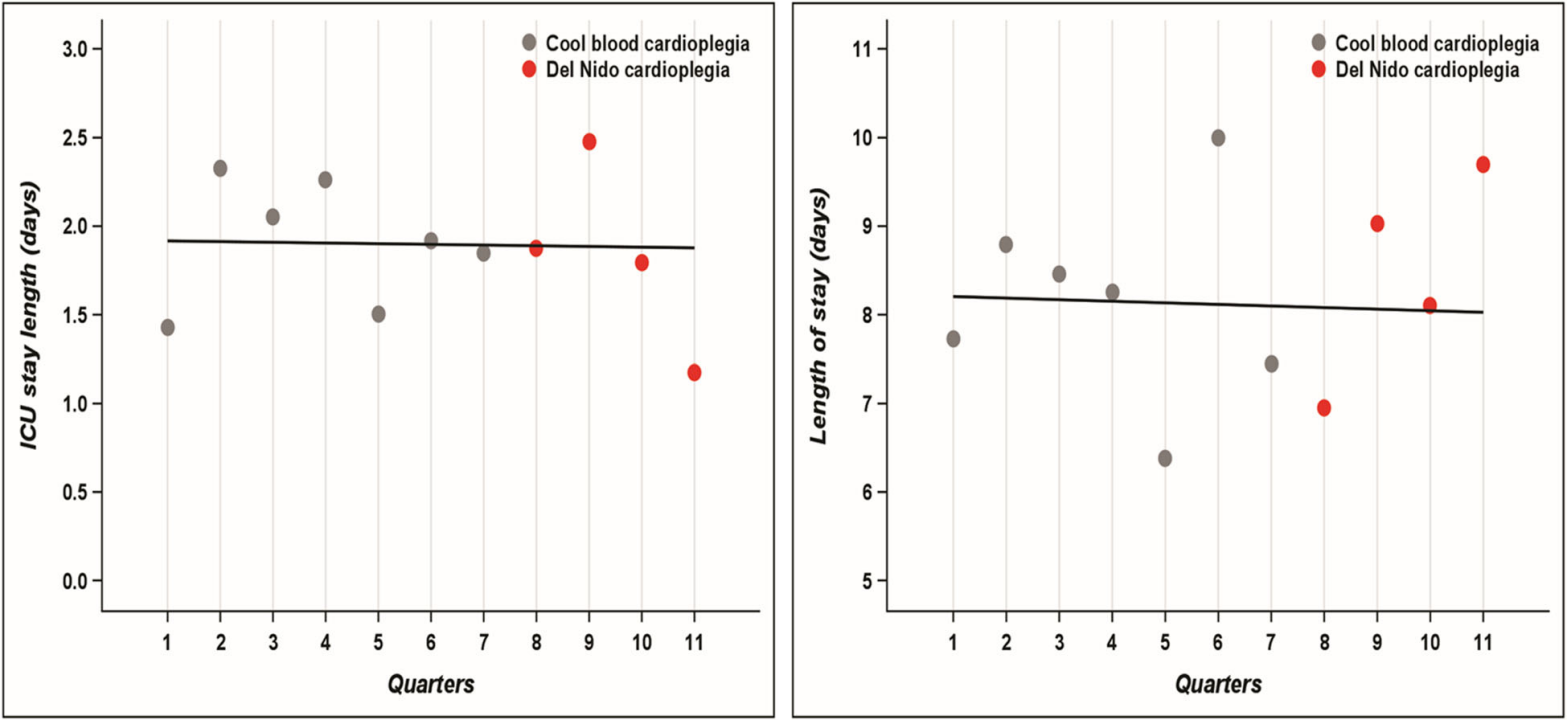
Linear regression of intensive care unit and hospital length of stays in days for cold blood cardioplegia obtained during 2015–2016 trimesters (quarters) to predict quarterly values for the 2016–2017 period

Postoperative outcomes are shown in Table 4. DN and CB groups had similar outcomes including ICU length of stay, ventilation length, hospital length of stay as well as occurrence of stroke, renal failure, sternal infection, gastrointestinal complication, hepatic dysfunction, bleeding, transfusion rate and mortality. A linear relation was obtained for the ICU and hospital length of stays when accounting for different trimesters of cardioplegia use (Fig. 1). The data distribution obtained with Del Nido cardioplegia matched the distribution of cold blood cardioplegia. This shows that the improvement is not due to a change in practice or the effect of time.

**Table 4:** Postoperative outcomes. ICU: intensive care unit; SD: standard deviation

		UNMATCHED			MATCHED	
	DN (n = 131) No.(%) or mean ± SD(MED)	CB (n = 251) No.(%) or mean ± SD(MED)	p value	DN (n = 130) % or mean ± SD	CB (n = 228) % or mean ± SD	p value
ICU stay (days)	1.9 ± 1.9	1.9 ± 2.5	0.91	1.9 ± 1.8	2.0 ± 2.1	0.64
Length of ventilation (h)	9.4 ± 27.3(4.4)	9.1 ± 19.6(4.3)	0.90	9.5 ± 27.4	8.9 ± 13.1	0.83
Intubation >48 h	4(3.1)	9(3.6)	1.0	3.1	3.6	0.83
Length of stay (days)	8.2 ± 5.7	8.1 ± 5.3	0.82	8.0 ± 5.4	8.5 ± 4.3	0.39
Stroke	3(2.3)	5(2.0)	1.0	2.3	3.7	0.52
Deep sternal wound infection	3(2.3)	2(0.8)	0.34	2.3	1.0	0.57
Dialysis	4(3.1)	5(2.0)	0.50	3.1	2.8	0.89
Gastrointestinal complication	5(3.8)	13(5.2)	0.62	3.1	6.6	0.20
Hepatic Dysfunction	3(2.3)	11(4.4)	0.40	2.3	5.8	0.17
Reoperation for Bleeding	8(6.1)	15(6.0)	1.0	6.2	4.7	0.61
In hospital transfusion	90(68.7)	173(68.9)	1.0	68.5	68.8	0.95
In hospital Mortality	3(2.3)	9(3.6)	0.76	2.3	5.1	0.24

### Myocardial protection

Myocardial protection outcomes are described in Tables 5 and 6. DN and CB groups had similar postoperative LVEF, needs for inotropic drugs for low cardiac output, atrial and ventricular arrhythmias, and conduction abnormalities (Table 5). Postoperative Troponin T levels evolved differently in time between the two groups but there was not statistically significant difference (Table 6).

**Table 5:** Myocardial protection outcomes

		UNMATCHED			MATCHED	
	DN (n = 131) No.(%) or mean ± SD	CB (n = 251) No.(%) or mean ± SD	p value	DN (n = 130) % or mean ± SD	CB (n = 228) % or mean ± SD	p value
LVEF (%)	52 ± 11	50 ± 12	0.22	52 ± 11	51 ± 10	0.37
Needing Inotropes for low cardiac outflow	11(8.4)	17(6.8)	0.54	8.5	9.2	0.84
Postop Atrial Flutter	34(26.0)	42(16.7)	0.04	25.4	19.0	0.43
Atrial Fibrillation de Novo	35(26.7)	74(29.5)	0.63	26.9	26.5	0.93
Postop VT/VF	8(6.1)	7(2.8)	0.16	5.4	4.1	0.63
Conduction Abnormalities	6(4.6)	13(5.2)	1.0	4.6	4.6	1.0
Pacemaker	5(3.8)	9(3.6)	1.0	3.9	4.5	0.80

*LVEF* Left ventricular ejection fraction, *VF* ventricular fibrillation, *VT* ventricular tachycardia

**Table 6:** T Troponin evolutions: post-operative values

		UNMATCHED			MATCHED	
T Troponin (ng/L)	DN (n = 131) mean ± SD	CB (n = 251) mean ± SD	p value	DN (n = 130) mean ± SD	CB (n = 228) mean ± SD	*p* value
Day 0	872 ± 1617	906 ± 1118	0.81	871 ± 1623	958 ± 854	0.50
Day 1	860 ± 1137	933 ± 1016	0.52	853 ± 1139	993 ± 823	0.21
Day 2	677 ± 1465	613 ± 686	0.57	671 ± 1469	636 ± 519	0.77
Day 4	450 ± 545	461 ± 483	0.84	442 ± 540	463 ± 317	0.67
Maximum peak	1186 ± 2116	1119 ± 1243	0.70	1182 ± 2124	1171 ± 957	0.95

## Discussion

The safeguard of cardiac surgery requires good myocardial protection and good “all other organ” protection. Intermittent cold blood cardioplegia is heart-safe but cumbersome. Minor changes in surgical flow and reduced times can impact mortality [5]. Del Nido cardioplegia has a safe record in pediatric cardiac surgery and has been used in many adult cardiac surgery centers for the last decade. Recent studies of selected adult population have demonstrated encouraging outcomes [6–18]. A transition from intermittent cold blood to Del Nido cardioplegia for all-comers seemed a potential way to improve surgical flow. The current study supports the routine use of Del Nido cardioplegia in all cases of adult cardiac surgery.

Myocardial protection concerns have been raised on the use of DN cardioplegia in adults and acquired cardiovascular disease patients. Use for diabetics with extensive microvascular disease and administration by a retrograde route have been debated [15, 16]. The growing body of literature tends to refute these concerns. Myocardial protection results in DN group are superimposable to the ones in cold blood cardioplegia even with a patient population composed of ~ 30% diabetics and ~ 20% with poor left ventricular function. Indeed, in the postoperative period both groups had a LVEF around 50%. Average Troponin T levels were lower but not statistically different unlike other reports [8, 14]. A recently published study by Kim et al. [9] compared levels of Troponin I and creatine-kinase-MB in patients undergoing cardiac surgery with Del Nido or cold blood cardioplegia and showed lower levels of both enzymatic markers in patients having received Del Nido cardioplegia. The use of Del Nido cardioplegia could be better for the aged heart as suggested by rodent-based studies [22, 23]. This study lends supports to the use of Del Nido cardioplegia in adults.

### Operative outcomes

Del Nido cardioplegia allows for minimal interruption during surgical procedures compared to cold blood cardioplegia. The mean aortic cross-clamp time in DN group requires no redosing. Taking into consideration the 2400 mL difference of total cardioplegia volume given between groups and an administration rate of 300 mL/min, Del Nido solution spares 8 min of aortic cross-clamp time for the cardioplegia delivery itself. The releasing/regaining exposure time may account for the other 13 min of aortic cross-clamp spared. Del Nido cardioplegia use allows for sequence modifications to optimise surgical flow. Altogether, this reflects a 20% shorter aortic cross-clamp time in DN group. Other studies showed similar results in terms of length of operative times [8, 14, 17]. Ad et al. published the only randomized controlled trial on the use of Del Nido cardioplegia in adult showing consistent results with these findings without reaching statistical significance [18]. The most important gain in terms of spared time comes from the procedures with the most steps such as for CABG and combined procedures (CABG + valves). Exploratory data on single and multiple valves (Table 3) suggests that there is the most benefit for multiple valves. This study is underpowered to prove so but it is likely that patients who would benefit the most from Del Nido cardioplegia would be the ones with more complex surgeries.

Interestingly, DN group had lower total cardioplegia volume and blood sugar level, although the clinical relevance of these findings remains inconclusive. The total Del Nido cardioplegia volume given was one third of the volume of cold blood cardioplegia but did not translate into fewer transfusions such as reported by Kim et al. [9]. It is possible that the reduction in cardioplegia volume in combination with the more physiologic nature of the cardioplegic solution may contribute to myocardial protection by reducing myocardial edema [24]. This is reported with the use of microplegia solutions [25]. Of possible benefit for diabetic patients, CPB peak glucose level was lower in DN group possibly allowing for easier blood glucose management during surgery [15]. Mick et al. reported similar findings when comparing Del Nido to Buckberg cardioplegia [10]. Glycemic control is a major concern in cardiac surgery. High glycemia is related to higher risk of complications such as mediastinitis while lower glycemia can lead to poor neurological control [15]. This study was not designed to conclude if a better glycemic control with Del Nido cardioplegia translates into a lower rate of these rare complications. A strict glucose management protocol was not used, although local clinical policies regarding glycemic control are well disseminated.

### Postoperative

Sorabella et al. [12] and Yerebakan et al. [14] studied Del Nido cardioplegia use in redo aortic valve surgery and isolated CABG after acute myocardial infarction, respectively; shorter in-hospital length of stay was reported. In this all-comer cohort, it is likely that this benefit can be offset by factors such as having one fifth of patients with preoperative LVEF < 50%. Interestingly, around one tenth of the patients in both groups had a LVEF < 35% for whom a mortality of 1% was found in CB group and no deaths in DN group. In this subset of patients, an increase in LVEF of ≥10% was seen postoperatively in 20% of patients from CB group and 40% of patients DN group, while no decrease in LVEF were found. Ideal myocardial protection strategy in this subgroup of patients is still to be found. Patients in both cardioplegia groups had similar surgical risks preoperatively as calculated by Parsonnet score and Euroscore II. This study reports no significant differences in term of complications between both cardioplegias and the observed mortality was lower than predicted by risks scores in both groups. This demonstrates that the absence of difference between the two groups is not based on patient selection or a learning curve effect. Similarly, the regression model used to predict the length of stay if cold blood cardioplegia was continued in time is superimposable to the actual data obtained with Del Nido cardioplegia. This supposes that the overall management of patients remains the same over the course of the study. Finally, even if the study was not powered to detect significant differences in rare events it is interesting to note that major gastrointestinal and hepatic complications appeared less frequent in DN group. These rare complications could be significantly decreased in larger cohorts of Del Nido cardioplegia with shorter CPB duration.

### Limitations

This study is limited by its retrospective, observational and single-surgeon single-institution design. The study includes heterogenous patients, although the proportion of types of procedures was matched. Although there is an historical bias with both cohorts being sequential, the clinical significance should be limited. From a clinical standpoint, lidocaine was administered in both groups since the “local cold blood recipe” contains lidocaine.

## Conclusions

Altogether, Del Nido cardioplegia appears safe for use in all adult cardiac surgery cases. The surgical strategy allowed by the use of Del Nido cardioplegia seems to confer adequate myocardial protection and all other organ protection. This strategy is associated with decreased operative times compared to intermittent cold-blood cardioplegia. Trials including large cohorts of patients with decreased ventricular function, diabetes and emergency surgeries are required.

## Data Availability

Complete data are available from the corresponding author upon request.

## Abbreviations

CABG: Coronary artery bypass grafting
CB: Cold blood
CPB: Cardiopulmonary bypass
DN: Del Nido
ICU: Intensive care unit
KCl: Potassium chloride
L: Liter
LV: Left ventricle
LVEF: Left ventricle ejection fraction
SD: Standard deviation
